# CMV Infection Is Directly Related to the Inflammatory Status in Chronic Heart Failure Patients

**DOI:** 10.3389/fimmu.2021.687582

**Published:** 2021-08-12

**Authors:** Alejandra García-Torre, Eva Bueno-García, Rocío López-Martínez, Beatriz Rioseras, Beatriz Díaz-Molina, José Luis Lambert, Covadonga Quirós, Sara Alonso-Álvarez, Rebeca Alonso-Arias, Marco A. Moro-García

**Affiliations:** ^1^Immunology Department, Hospital Universitario Central de Asturias, Oviedo, Spain; ^2^Department of Cardiac Pathology, Health Research Institute of the Principality of Asturias – ISPA, Oviedo, Spain; ^3^Laboratory Medicine Department, Hospital Universitario Central de Asturias, Oviedo, Spain; ^4^Section of Hemodynamics and Interventional Cardiology, Department of Cardiology, Hospital Universitario Central de Asturias, Oviedo, Spain; ^5^Clinical Biochemistry Department, Hospital Universitario Central de Asturias, Oviedo, Spain; ^6^Hematology and Haemotherapy Department, Hospital Universitario Central de Asturias, Oviedo, Spain

**Keywords:** CHF, CMV, inflammation, T-lymphocyte, immunosenescence, TNF, IL-6

## Abstract

High levels of inflammation play an important role in chronic heart failure (CHF). Patients with CHF have elevated levels of pro-inflammatory cytokines circulating systemically, mainly TNF and IL-6. However, there are almost no studies that relate these levels to the functional status of patients in CHF, much less to their CMV serostatus. In this study, patients with CHF (n=40; age=54.9 ± 6.3; New York Heart Association functional classification (NYHA, I-III) and healthy controls (n=40; age=53.5 ± 7.1) were analyzed. The serum concentrations of nine pro- and anti-inflammatory cytokines were measured by Luminex^®^ xMap Technology and the basal level of mRNA expression of some immune molecules was quantified by TaqMan™ Array in CD4+ T-lymphocytes. The concentration of these cytokines in culture supernatants in response to anti-CD3 and LPS was also measured. The percentage of CD28null T-cells was determined, as well as the antibody titer against CMV. We found a higher concentration of all cytokines studied in CHF serum compared to healthy controls, as well as a direct correlation between functional status in CHF patients and levels of inflammatory cytokines. Moreover, the highest cytokine concentrations were found in patients with higher concentrations of lymphocytes lacking CD28 molecule. The cytokine production was much higher in CMV+ patients, and the production of these cytokines was found mainly in the T-lymphocytes of CMV+ patients in response to anti-CD3. Anti-CMV antibody levels were positively correlated with cytokine levels. The baseline expression of specific mRNA of the main molecules involved in the Th1 response, as well as molecules related to the CD4+CD28 null subset was higher in CMV+ patients. The cytokine concentrations are higher in CHF CMV+ patients and these concentrations are related to the production of antibodies against CMV. These high levels of cytokines are also associated with the more differentiated CD28null lymphocyte populations. All this, together with the dynamics of the pathology itself, makes CMV+ patients present a worse functional status and possibly a worse evolution of the pathology.

## Introduction

The process known as immunosenescence may affect both the elderly and individuals of all ages with chronic inflammatory or infectious diseases. The changes produced by immunosenescence are therefore found in patients with chronic heart failure (CHF). The immunosenescence found in CHF patients is not only associated with the pathology itself but also with a worse functional status (1). The aging of the immune system, mainly adaptive, has been associated with the presence of chronic and persistent antigens, as well as a low-level inflammatory state, maintained for a considerable period of time. All these processes lead to a dysregulation of the immune system, compromising immune responses, producing an increase in the frequency of highly differentiated T-lymphocytes, mainly with the loss of the CD28 molecule (2, 3).

The inflammation found in patients with CHF may be a consequence of the increase in the pro-inflammatory cytokines expression as mediators of the protective effect on cardiac cells, as a rapid adaptation to the stress suffered by these cells (4). On the other hand, this increase in pro-inflammatory cytokines leads to an advance in cardiac pathology due to the harmful effect that these cytokine present on the heart-cells and on the systemic circulation (5, 6). IFN-γ production, mainly by CD4+ Th1 lymphocytes, is directly related to these deleterious effects produced by circulating pro-inflammatory cytokines (7–9). Accordingly, the differentiation of T-lymphocytes and the increase in the concentration of pro-inflammatory cytokines in physiological aging, and in certain chronic diseases, are events that occur at the same time. Because of this it is not at all clear what produces what. Differentiated T-lymphocytes are related to the production of inflammatory cytokines, while a high concentration of circulating cytokines has been related to the differentiation of T-lymphocytes.

Not much is known about the antigens involved in the differentiation of T-lymphocytes in the immunosenescence process in the context of CHF. T-lymphocytes could be activated repeatedly and continuously over time by antigens from chronic infections, and this continuous activation could be the cause of increased inflammatory degree and probable tissue damage. To date, the main known inducer of T-cell differentiation is CMV. This virus has been related to the immunosenescence process, even the antibody titer against CMV has been related to immunocompetence, and the degree of lymphocyte differentiation in the elderly (10–14). Recent studies in our laboratory have also found this association in patients with CHF (15).

The objective of this study was to investigate the implication of CMV infection in the production of pro-inflammatory cytokine, and its relationship with functional status in CHF patients.

## Materials and Methods

### Study Population

Forty healthy volunteers and 40 chronic heart failure (CHF) patients were recruited for the study. Individuals in the study were divided into two groups: healthy control (n=40) and CHF patients (n=40). In turn, each of these groups was divided according to their CMV-seropositivity (**Table 1**). All volunteers were defined as individuals younger than 65 years old to reduce the effect of aging on the study. The control group was recruited from the Centro de Transfusiones del Principado de Asturias (Oviedo, Spain). CHF patients were classified according to the New York Heart Association functional classification (NYHA) and recruited from the Heart Failure Unit at Hospital Universitario Central de Asturias with symptomatic HF (NYHA class I to III). All subjects underwent a physical examination and answered a standardized questionnaire to assess their medical history, current illnesses, and any medication they were taking. Exclusion criteria included all conditions that might influence the immune system, such as a recent or current infection, autoimmune disease or tumor, malnutrition, abnormal laboratory data (hemoglobin < 12 g/dL, leukopenia < 3500 cells/µL, neutropenia < 1500 cells/µL, leukocytosis > 15000 cells/µL and platelets < 105 cells/µL), and pharmacological interference. Informed consent was obtained from all volunteers before participation in the study. The study was approved by the ethics committee of the Hospital Central de Asturias (Oviedo, Spain) with the number 82/17. Peripheral blood samples were drawn from all subjects for hematological and immunological analyses.

**Table 1:** Participant characteristics in relation to CMV serostatus.

	Chronic Heart Failure Patients		Healthy Controls		ANOVA		
	(CHF) (n=40)		(HYC) (n=40)				
	CMV-	CMV+	CMV-	CMV+	P_pathology_	p_CMV_	p_interaction_
	(n=13)	(n=27)	(n=19)	(n=21)			
**Demographic data**							
Age ± SD	53.1 ± 8.9	56.7 ± 5.6	52.1 ± 7.5	54.9 ± 6.6	NS	NS	NS
Male (%)	10 (76.9)	22 (81.7)	16 (84.2)	13 (61.9)	NS	NS	NS
BMI ± SD (kg/m^2^)	23.9 ± 3.6	24.4 ± 2.8	23.8 ± 3.6	25.0 ± 2.9	NS	NS	NS
Smoking status, current (%)	4 (30.8)	6 (22.2)	4 (21.1)	5 (23.8)	NS	NS	NS
Hypertension (%)	2 (15.4)	5 (18.5)	0 (0)	0 (0)		NA	
% LVEF ± SD	37.2 ± 15.4	36.1 ± 10.6	>60%	>60%		NA	
Diabetes mellitus (%)	3 (23.1)	6 (22.2)	0 (0)	0 (0)		NA	
Cholesterol ± SD (mg/dL)	173.1 ± 38.1	152.3 ± 25.5	ND	ND		NA	
NT-proBNP, pg/mL (IQR)	4985 (4759)	5304 (4172)	ND	ND		NA	
CRP, mg/dL (IQR)	1.6 (1.65)	1.6 (2.75)	ND	ND		NA	
**CHF etiology**							
Coronary artery disease (%)	5 (38.5)	12 (44.4)	NA	NA		NA	
Idiopathic dilated cardiomyopathy (%)	5 (38.5)	9 (33.3)	NA	NA		NA	
Others (%)	3 (23.1)	6 (22.2)	NA	NA		NA	
**Hematological variables (mean and SD)**							
WBCs (10^3^/µl)	7.7 ± 1.9	8.2 ± 1.9	6.4 ± 1.8	6.9 ± 1.8	0.007	NS	NS
Neutrophils (10^3^/µl)	5.0 ± 1.7	5.4 ± 1.8	3.9 ± 1.0	4.3 ± 1.0	0.025	NS	NS
Neutrophils (%)	64.1 ± 8.9	64.1 ± 8.1	54.2 ± 8.7	56.4 ± 6.6	0.001	NS	NS
Monocytes (10^3^/µl)	0.7 ± 0.2	0.6 ± 0.1	0.5 ± 0.3	0.4 ± 0.2	0.002	NS	NS
Monocytes (%)	8.9 ± 2.6	8.2 ± 2.5	7.0 ± 4.4	5.7 ± 2.3	0.009	NS	NS
Lymphocytes (10^3^/µl)	1.7 ± 0.7	1.9 ± 0.6	2.6 ± 0.7	3.2 ± 0.7	<0.001	NS	NS
Lymphocytes (%)	23.3 ± 8.8	24.3 ± 7.2	36.2 ± 3.8	41.6 ± 5.0	<0.001	NS	NS

LVEF, left ventricular ejection fraction; NT-proBNP, N-terminal protype B natriuretic peptide; BMI, body mass index; CRP, C-reactive protein; WBCs, white blood cells; SD, standard deviation; IQR, interquartile range; NA, not applicable; ND, not done; NS, not significant.

### Hematological Analysis and Immunological Phenotyping

The hematological parameters were determined using a Sysmex XT-2000i (Sysmex, Hamburg-Norderstedt, Germany), and the biochemistry values using a Cobas c711 analyzer series (Roche Diagnostics, Indianapolis, USA). For flow cytometry analysis, peripheral blood cells were surface-stained with anti-CD4 (PerCP), anti-CD8 (PE), anti-CD3 (FITC) and anti-CD28 (APC) (Biolegend, San Diego, CA, USA). One hundred microliters of whole blood from volunteers were stained with the labeled monoclonal antibodies for 20 min at room temperature. Samples were red-blood lysed with FACS Lysing Solution (BD Biosciences), washed in PBS, and analyzed using Kaluza software in a Gallios cytometer (Beckman-Coulter, Brea, CA, USA). Appropriate isotype control mAbs were used for marker settings.

### Cytomegalovirus Serology

Serum presence of CMV-specific antibodies was determined by an enzyme-linked immunosorbent assay, Vir-ELISA Anti-CMV-IgG (Viro-Immun Labor-Diagnostika GmbH, Oberursel, Germany), according to the manufacturer’s specifications. CMV-serostatus was interpreted by means of the calculation of the ratio: Cut-off Index = optical density (OD) value of sample / cut-off value, whereby a ratio of 1.0 is equivalent to the cut-off value. Cut-off indexes >1.1 were considered positive. Quantification of anti-CMV antibody titers was performed through a semi-quantitative titer calculation.

### Isolation of PBMC and Cell Cultures

Peripheral blood mononuclear cells were isolated from peripheral blood that had been anticoagulated with EDTA by centrifugation on Ficoll-Hypaque gradients (Lymphoprep; Nycomed, Oslo, Norway). Cultures were performed in RPMI 1640 medium containing 2x10-3 M L-glutamine and Hepes (BioWhitaker, Verviers, Belgium) and supplemented with 10% FCS (ICN Flow; Costa Mesa, CA, USA) and antibiotics. Cells were incubated at 37°C and 5% carbon dioxide.

### Cytokine Quantification in Patient Serum and Supernatants

The sera of the individuals under study were collected and stored at -80°C until the cytokine quantification and analysis. Meanwhile, response to anti-CD3 (1 μg/mL) (eBioscience, San Diego, CA, USA) and to LPS (1 μg/mL) was analyzed in PBMCs (2x10^6^ cells/ml) from CHF patients. PBMCs were cultivated alone or stimulated with anti-CD3 and LPS in 48-well plaques in a humidified 37°C incubator for three days. Finally, cell-free supernatants were collected and stored at −80°C for multiplexed cytokine analyses.

The production of 9 different cytokines (IFN-γ, IL-10, IL-12, IL-17, IL-1β, IL-2, IL-4, IL-6 and TNF) was quantified in sera and supernatants using the ProcartaPlex™ Mix & Match Panel (Affymetrix eBioscience, San Diego, USA) and the Luminex^®^ xMap Technology (Luminex Corporation, Austin, USA) equipment following manufacturer’s settings.

### Cytokine Expression Array

To isolate CD4+ T-cells, PBMCs from 5 CMV-positive and 5 CMV-negative CHF patients were isolated by centrifugation on Ficoll-Hypaque gradients (Lymphoprep; Nycomed, Oslo, Norway) after 20 min of incubation with the RosetteSep Human CD4+ T-cell Enrichment Cocktail (StemCell Technologies, Grenoble, France). In all cases, purity of isolated CD4+ T-lymphocytes, tested by flow cytometry was higher than 95%. mRNA was extracted using a Total RNA Isolation (Macherey-Nagel GmbH & CoKG, Düren, Germany) according to the manufacturer’s instructions. Reverse transcription of mRNA isolated from each sample was carried out in a 20 µL final volume with the iScript cDNA Synthesis Kit (Bio-Rad, Life Science Research Group, Hercules, CA, USA) following manufacturer’s instructions. The mixture was incubated at 25°C for 5 min, at 42°C for 30 min, and at 85°C for 5 min and stored at -80°C until required for the array. Equal quantities of cDNA were mixed to generate two pools, one with samples from CMV-seronegative patients and another one with samples from CMV-seropositive patients. Cytokine gene expression was examined through TaqMan™ Array Human Immune Response Real-Time PCR (Applied Biosystems, Foster City, CA, USA) using predesigned human gene-specific primers and probes based on published cytokine sequences and following manufacturer’s instructions.

### Statistical Analysis

Results are expressed as the median and interquartile range (IR) or the mean and standard deviation. Quantitative variables were compared using the analysis of variance (ANOVA) to study the effect of CMV (CMV- or CMV+), pathology (CHF+ or CHF-) or NYHA (Class I+II or Class III) and adjusting for sex. If significant interactions were observed in any of these analyses, comparisons with a Bonferroni-correlated post-hoc test were performed. In order to perform these analyses, non-parametric variables were normalized by logarithmic transformation. Groups were compared using the non-parametric Mann-Whitney U test (for non-normally distributed data) or Student’s t-test (for normally distributed data). To compare the results obtained in the expression arrays, we used the comparative ddCT method (16) to calculate relative quantitation of gene expression after outlier removal and data normalization based on the endogenous control genes expression (18S rRNA, GAPDH, HPRT1 and GUSB) using DataAssist software (Thermo Fisher Scientific). The list of analyzed genes and their assay IDs is presented in the **Supplementary File** (**Supplementary Table 1**). The outlier and the extreme values were calculated by adding 1.5 and 3 times the interquartile range (IR) to the 75th percentile, respectively. Correlations between variables were assessed using the non-parametric Spearman test (ρ). Analyses were performed using the PASW Statics 17.0 statistical software package (IBM SPSS, NY, USA) and p-values of 0.05 or less were considered significant.

## Results

### Characteristics of Studied Groups Related to CMV-Serostatus and Cytokine Levels

In **Table 1** we can see the characteristics of the two studied groups, CHF patients and healthy control group (HC). All the participants in the study belong to the Caucasian ethnic group. Levels of the antibodies against CMV were measured in all participants. All study volunteers had a blood test and immunophenotype. When we made statistical comparisons with the ANOVA test, we found significantly higher levels in total white blood cells (WBCs), monocytes, and neutrophils and significantly decreased levels in lymphocytes (**Table 1**). We did not find any other difference in relation to CMV serostatus in any leukocyte subpopulation or in any other measured variable.

**Figure 1:**
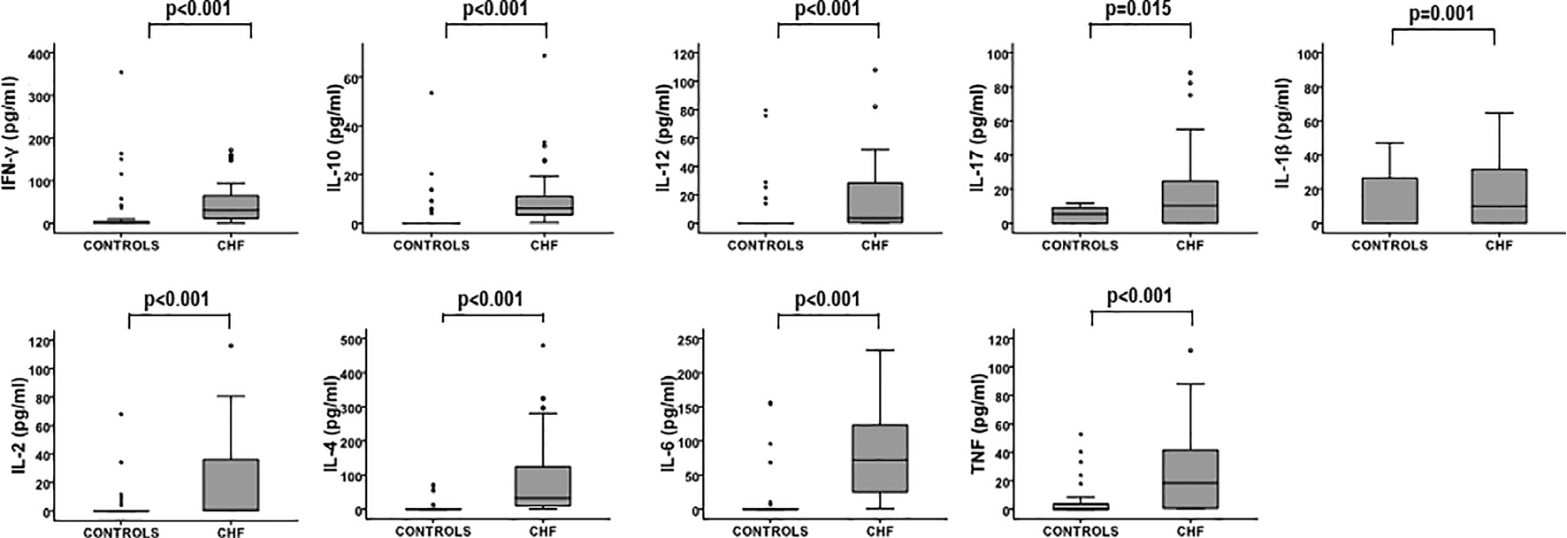
Cytokine levels in the two studied groups. Cytokine concentrations were measured using Luminex multiplex technology. Levels of cytokines in CHF (n = 40) and controls (n = 40) are illustrated in the box plots. Differences between groups in the levels of cytokines were compared using the Mann-Whitney Test, p-values are depicted in the panels. Outlier values are represented by circles and extreme values by stars, calculated by adding 1.5 and 3 times the IR to the 75th percentile, respectively.

After measuring the cytokine concentrations of pro-inflammatory cytokines (IFN-γ, IL-12, IL-17, IL-1β, IL-2, TNF), anti-inflammatory (IL-10 and IL-4), and the pro- and anti-inflammatory cytokine IL -6, in the CHF group and controls, we found that the levels of cytokines were always significantly higher in the CHF group (Mann-Whitney Test, p < 0.001 in all cases except IL-17, p=0.015, and IL-1β, p=0.001) (**Figure 1**). In addition to the increased levels of the cytokines studied in CHF patients, all of them were positively correlated with each other (**Supplementary Table 2**). Therefore, in some of the figures we will only display the most representative cytokines, the rest of the figures of the cytokines can be consulted in the **Supplementary Material**.

Despite CHF patients showing higher CMV infection rate, total WBCs and leukocyte populations are related only to pathology and not to CMV serostatus in the groups studied. The levels of the cytokines studied are clearly increased in patients with CHF and all of them are correlated with each other.

### Association Between CMV-Serostatus and Level of Cytokines in CHF

**Figure 2:**
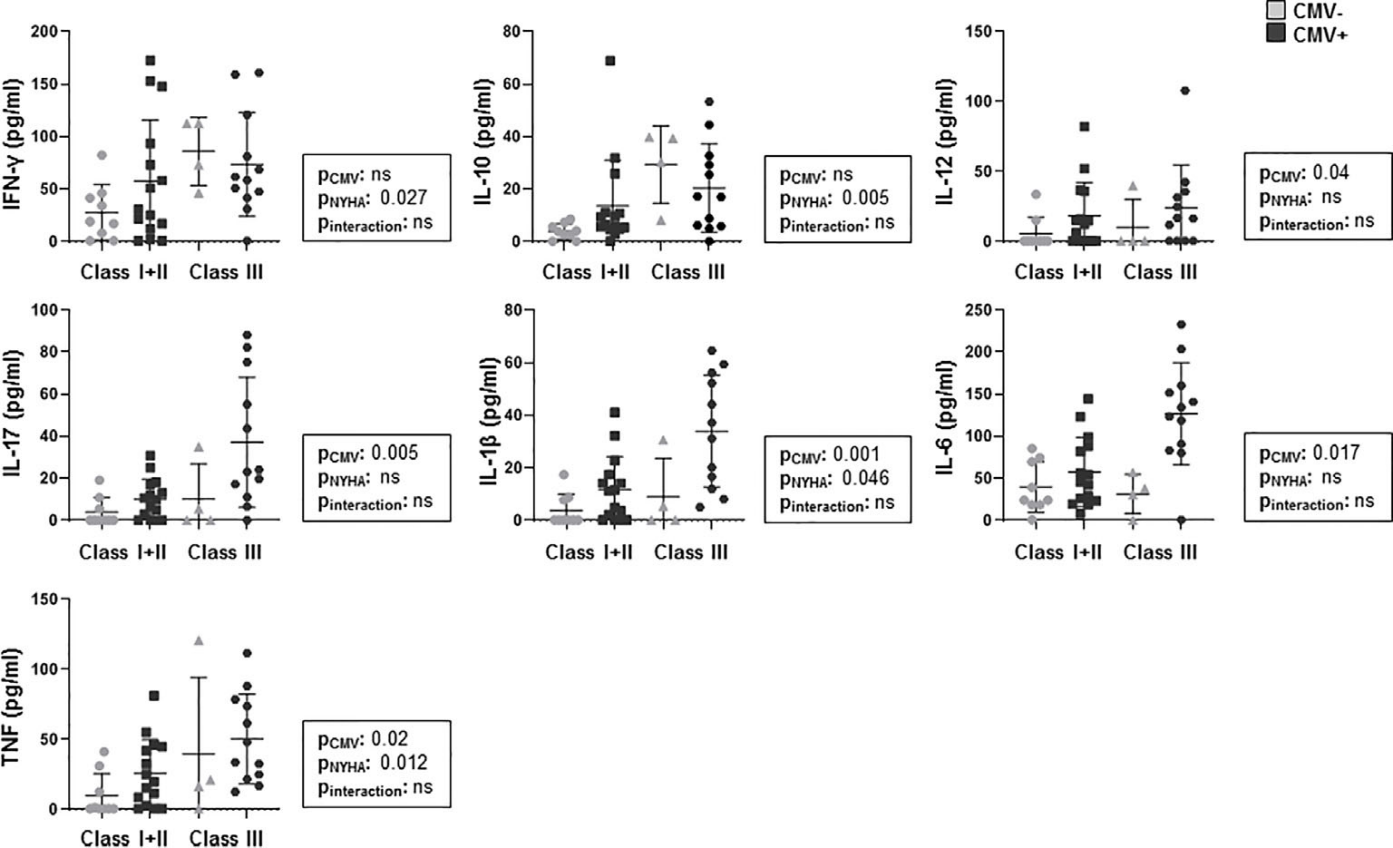
Levels of cytokines analyzed in CHF with respect to the different degrees of HF classified according NYHA (New York Heart Association) in CMV- and CMV+ patients. Outlier values are represented by circles and extreme values by stars, calculated by adding 1.5 and 3 times the IR to the 75th percentile, respectively. The ANOVA test was used to examine differences between the groups; p-values are depicted in the boxed text; the interaction is between NYHA and CMV serostatus. If significant interactions were observed, comparisons with a Bonferroni correlated *post hoc* test were performed and p-values are represented in the panels.

To evaluate the association of CMV infection with cytokine production we divided our CHF patients according to their CMV serostatus, 27 out of 40 were CMV-seropositive. Moreover, we classified the patients according to the extent of CHF by functional criteria (NYHA). As we only had three NYHA class I patients, we decided to combine the patients from groups I and II. We observed that the highest concentrations of cytokines were found in CMV-positive individuals from NYHA class III. We found that the cytokine concentrations were increased in the CMV+ patients in the case of IL-12, IL-17 and IL-6. These concentrations were also increased in CMV+ patients and belonging to NYHA class III in the case of IL-1β and TNF. When we analyzed IFN-γ and IL-10, we saw that their increase was only related to NYHA class III, but not to their CMV serostatus. In the case of IL-2 and IL-4, we did not find significant differences in patients with respect to their CMV serostatus or their functional class (ANOVA test, p<0.05) (**Figure 2** and **Supplementary Figure 1**).

**Figure 3:**
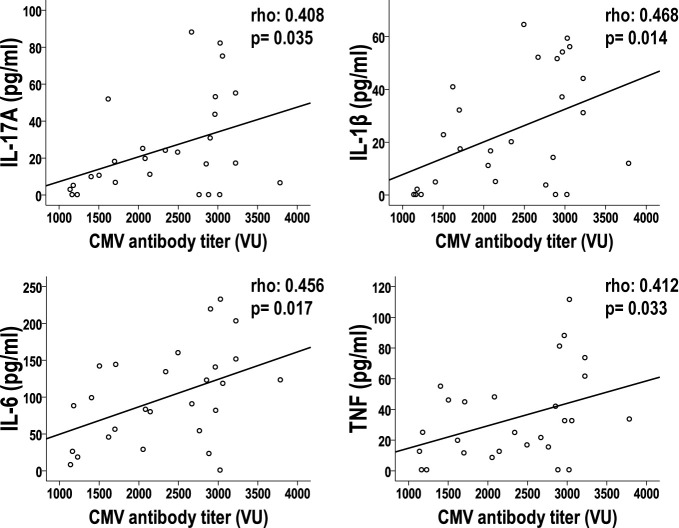
Relationship between anti-CMV antibody titer with cytokine levels in CHF patients. The correlation of anti-CMV antibody titers and cytokine levels in CHF patients is represented in the dot plots. Spearman’s test was applied to calculate the correlations; the p-value and coefficient of correlation are listed on the panels.

Next, we wanted to find out if there was any type of correlation between the antibody titer against CMV and the level of the cytokines analyzed. Levels of anti-CMV antibodies in CHF individuals were measured and they showed a median concentration of 2,336 VU/ml (IR, 1,350 VI/ml). We found a clear correlation between the levels of the cytokines IL-17, IL-1β, IL-6 and TNF and the antibody titer against CMV (Spearman Rho test; p<0.05) (**Figure 3**), the antibody titer is positively correlated with the levels of these pro-inflammatory cytokines. In the case of the rest of cytokines, we did not find a significant relationship, but a clear trend (**Supplementary Figure 2**).

In summary, we can confirm that the increase in cytokine concentrations in CHF patients is related both to the worsening of the functional status of the patients and to their serostatus against CMV, and these levels of cytokines correlate with the levels of antibodies to CMV.

### Relationship Between Cytokine Level and T-Lymphocyte Differentiation in CHF Patients

**Figure 4:**
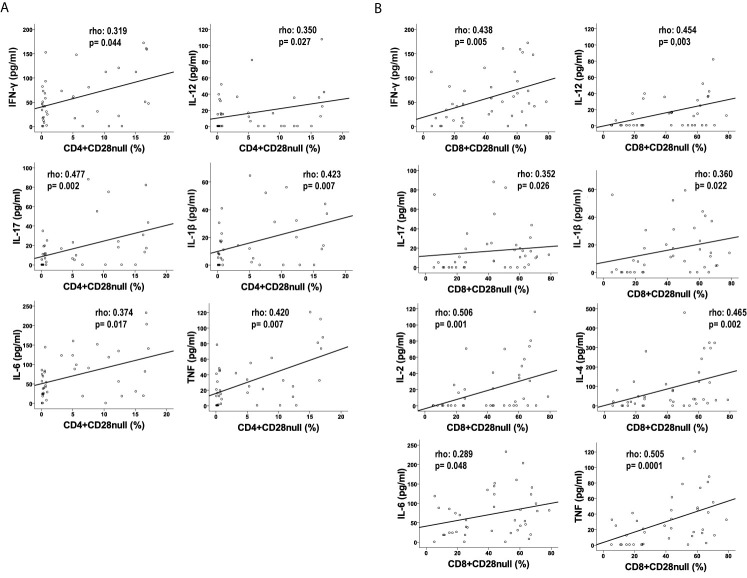
Levels of cytokines correlated to CD28null T-lymphocyte populations in CHF patients. Levels of cytokines (pg/mL) in CD4+ **(A)** and CD8+ T-lymphocytes **(B)**. Spearman’s test was applied to calculate the correlations; the p-value and coefficient of correlation are listed on the panels.

It is well known that the degree of differentiation in T-lymphocytes in the elderly is related to, among other things, an increase of a low basal inflammation or “inflammaging”. We observed more differentiated phenotypes in T-cells in CHF patients related to the concentrations of IL-6 (1). T-lymphocytes can be separated into less differentiated subsets that express the CD28+ marker and the most differentiated subsets with loss of expression of CD28. We wanted to verify the association between the levels of the studied cytokines and the T-cell differentiation in the CHF patients. For this, we face the percentage of CD4+CD28null T-cells and the level of cytokines in the CHF patients and we found a significant positive correlation between all the studied cytokines (Spearman Rho test; p<0.05), less in the case of IL-10, IL-2 and IL-4 where we did not find a significant correlation but a very marked trend in the case of IL-2 and IL-4 (**Figure 4A** and **Supplementary Figure 3A**). After analyzing the CD8+ T-lymphocyte populations, we also observed a strong correlation between all the studied cytokines (Spearman Rho test; p<0.05), but not in the case of IL-10. In patients with a larger CD8+CD28null population we found significant higher concentrations of all cytokines except for IL-10 (**Figure 4B** and **Supplementary Figure 3B**).

**Figure 5:**
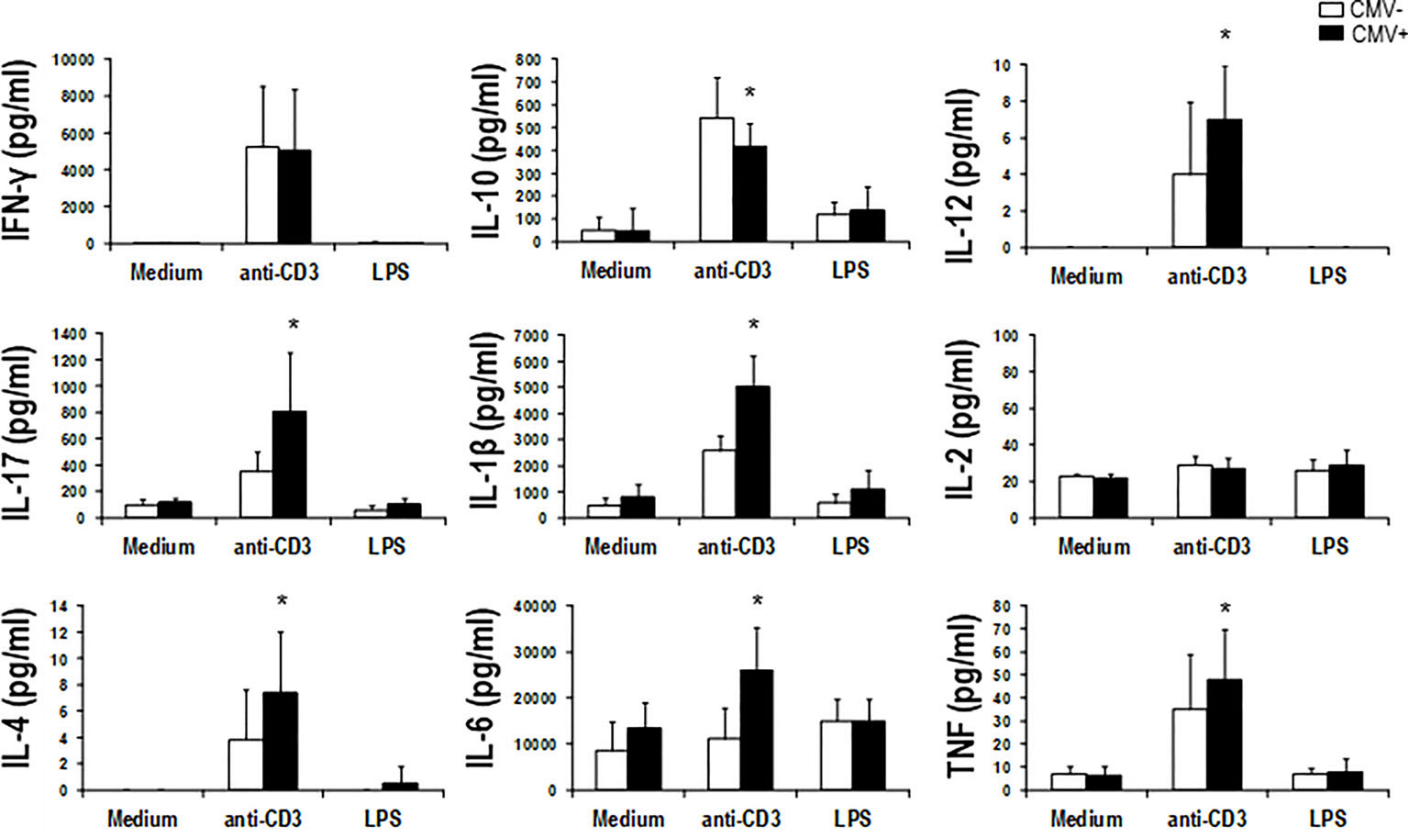
Cytokine levels produced in response to different culture conditions in CHF patients according to their CMV-serostatus. PBMCs from CHF patients (CMV-, n = 7; CMV+, n = 9) were cultured for 5 days in medium alone, in medium containing anti-CD3 (1 µg/ml), or in medium containing LPS (1 µg/ml). In the supernatant of the different cultures, the concentrations of the different cytokines were measured using Luminex multiplex technology and analyzed according their CMV-serostatus. Bar graphs summarize the concentrations of the cytokines in the supernatants of the cultures with the different conditions studied. (means ± SEM) from the studied subjects. Paired t-test was used to compare paired means, and p-values are depicted in the panels. *indicates a significant difference (p <0.05) compared to the other groups.

To test the implication of T-lymphocytes in pro-inflammatory cytokine production in CHF patients, we cultivated PBL’s alone, in the presence of anti-CD3, and in the presence of LPS. We only found a significantly high production of cytokines compared to the culture without stimulation in the case of anti-CD3 (Student’s t-test for paired data, p<0.001 in all cases except, curiously, IL-2). This could be indicating that most of these cytokines are being produced by T-lymphocytes. When we divide these results by CMV seropositivity, a significantly high production of cytokines is observed in all cases by CMV+ patients, except with anti-inflammatory cytokine IL- 10, where the highest production is observed in CMV- patients (Student’s t-test for paired data, p <0.05 in all cases except IFN-γ and IL-2) (**Figure 5**).

In summary, CD4+ and CD8+ phenotype is associated with the level of the cytokines; more differentiated CD4+ and CD8+ T-lymphocyte subsets are increased in CHF patients with higher pro-inflammatory cytokine levels. Furthermore, the production of these cytokines comes primarily from the T-lymphocyte population, with increased production in CMV+ patients.

### Differential mRNA Expression in CMV- and CMV+ Patients

**Figure 6:**
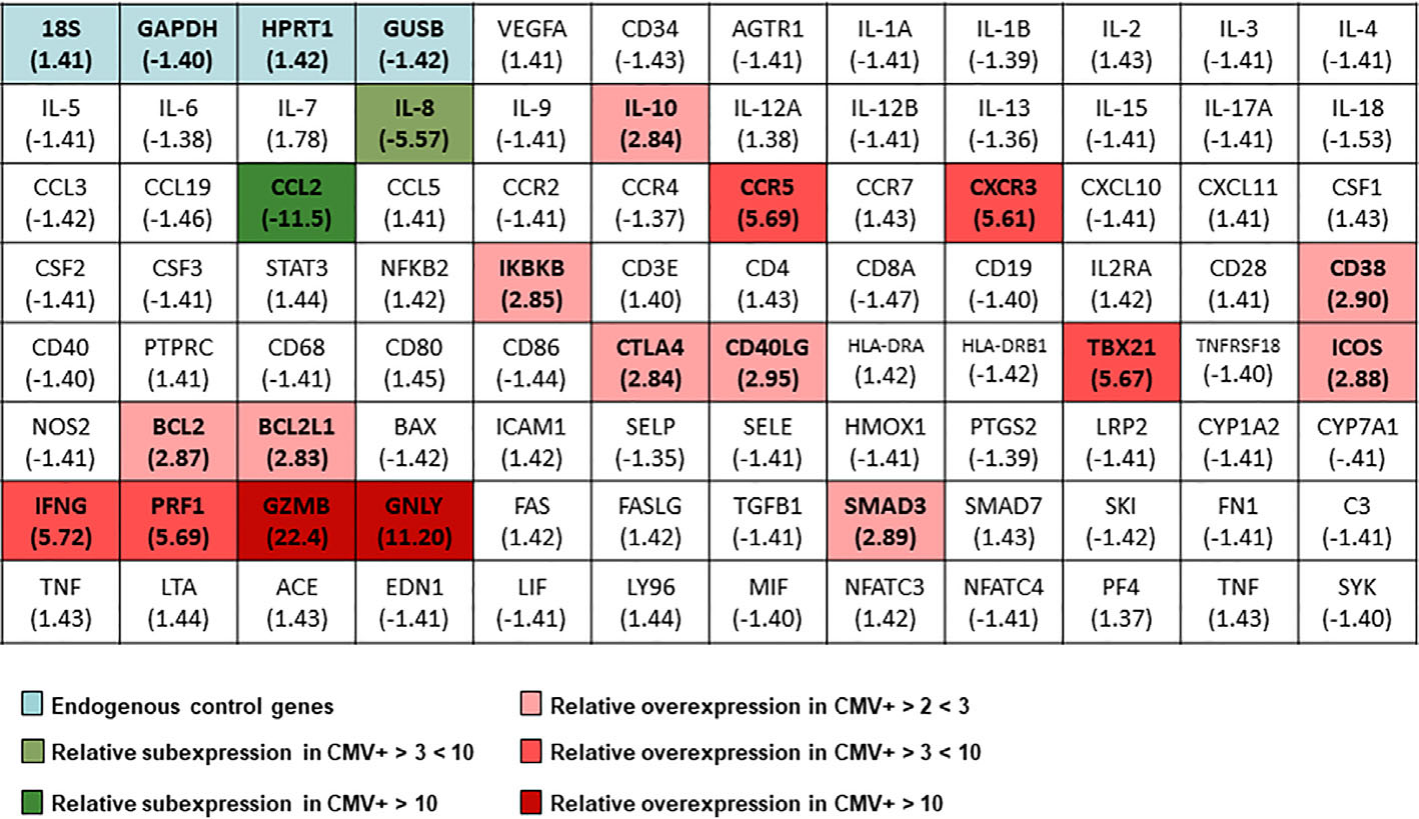
Changes in the gene expression profile in CHF patients divided according to their seropositivity to CMV measured by TaqMan™ Array Human Immune Response. Heat map showing differential expression in patients with CHF CMV+ compared to CMV- patients. Green colors indicate under-expression in CMV+ patients and red tones overexpression in these patients. The blue color is indicating the genes used as endogenous control genes in 5 CHF CMV- and 5 CHF CMV+ patients. We used the comparative ddCT method for calculating relative quantitation of gene expression after outlier removal and data normalization based on the endogenous control genes expression using DataAssist software (Thermo Fisher Scientific).

As it is already well known, the production of IFN-γ by the Th1 CD4+ T-lymphocytes are major contributors to heart failure (9). For this, we wanted to verify their influence in the high cytokine levels found in CHF patients. To this, we analyzed CD4+ baseline level of gene expression related to inflammation. We used TaqMan™ Array Human Immune Response plates where we measured separately the level of mRNA expression in a pool of 5 CMV- seronegative and 5 CMV-seropositive patients (**Figure 6**). We separated CD4+ lymphocytes, extracted their mRNA and quantified it in expression plate arrays.

After analyzing the results, we found that only two genes were under-expressed in the CHF CMV+ patients, IL-8 and CCL2, both involved in pro-inflammatory and chemotactic processes. A large number of genes were found to be overexpressed in CMV+ patients, and these genes can be categorized in some groups. We found IL-10 overexpressed, possibly as a reaction to the higher concentration of circulating pro-inflammatory cytokines that CMV+ patients present. We also found two of the main genes related to the Th1 cell response overexpressed, in this case TBX21 (T-bet) and IFN-γ. CD4+CD28null T-cells are associated with high cell differentiation and are found in a much higher proportion in CHF CMV+ patients (15). In addition, they present a cytotoxicity similar to CD8+ T-cells or NK cells and we found that the molecules related to this cytotoxicity are overexpressed, in this case, perforin, granzyme B and granulysin. We also observed overexpressed genes for molecules that are increased in activated CD4+ T-lymphocytes such as CD38, CTLA4, CD40LG, ICOS, CXCR3 and CCR5. Finally, the antiapoptotic genes BCL2 and BCL2L1 are also overexpressed in the CD4+ T-lymphocytes in CMV+ patients. Two other overexpressed genes were the SMAD3 genes, involved in the TGF-β production cascade, and the IKBKB gene, an inhibitor of the NF-κβ pathway.

In summary, we can say that CHF CMV+ patients present overexpressed genes of the Th1 pathway, molecules involved in the cytotoxicity of CD4+CD28null cells, molecules related to the activation of CD4+ T-cells and antiapoptotic molecules.

## Discussion

The present study exhibits that CHF patients show a higher level of cytokines than age-matched healthy controls, and this high level of cytokines is even higher in CMV+ CHF patients and in those with worse functional status. Moreover, not only seropositivity but also serum titers of anti-CMV antibodies are related to a higher level of proinflammatory cytokines such as IL-17, TNF, IL-1β and IL-6. CHF patients may display immunocompromised responses, resulting in an inability in controlling viral reactivations where CMV may be exacerbating T-cell differentiation, being these populations, the main producers of the cytokines analyzed in this study.

CHF is a disease with high morbidity and mortality, despite the treatments that have emerged in recent years, which seems to indicate that the pathogenic mechanisms are not fully controlled by these treatments. Permanent inflammation may be one of these underlying mechanisms unaltered by current treatments (17, 18). After the finding of elevated levels of TNF in sera from patients with CHF, it was seen that other pro-inflammatory cytokines are also elevated (19). Since then, numerous evidences have pointed to the activation of inflammatory pathways as an important pathological event in the onset and progression of the syndrome (20–22). The increased concentration of pro-inflammatory cytokines in the serum of patients with CHF compared to healthy controls is a circumstance that can be caused by some relevant processes in the context of CHF, among others: global aging, metabolic syndrome, chronic obstructive pulmonary disease (COPD), chronic kidney disease (CKD), atrial fibrillation and neurohormonal hypothesis of renin-angiotensin-aldosterone system (23–26). Our results have shown that chronic CMV infection is a main factor related to this inflammatory status.

The relationship between poorer functional status, (measured as NYHA) and the level of pro-inflammatory cytokines, mainly with TNF and IFN-γ, had already been demonstrated in other studies (27–29). This relationship has even been correlated with increased mortality (30) but it had never been demonstrated in relation to CMV infection in CHF, although the role of CMV as a marker of disease severity in acute heart failure had been described and its possible implication in the development and worsening of other cardiac pathologies has been seen (31–33). What has never been proven is the relationship that we have shown between poorer functional status, levels of inflammatory cytokines, and CMV seropositivity. Seropositive patients have a higher concentration of pro-inflammatory cytokines, and the levels of antibodies against CMV are directly correlated with the level of inflammatory cytokines. In view of these results, lowering the levels of pro-inflammatory cytokines in CHF could improve quality of life. In this way, administration of methotrexate, with its anti-inflammatory effects, has demonstrated improving NYHA (34). In view of our results, the possible vaccination against CMV at an early age or once the disease is diagnosed, could also be a strategy to improve functional status in CHF patients (35, 36).

Like the concentration of pro-inflammatory cytokines, we also found elevated concentrations of the two anti-inflammatory cytokines studied, IL-10 and IL-4, contrary to what was found in another study from the 2000s (37). This elevation could be justified in the context of an environment with high concentrations of pro-inflammatory cytokines, in which IL-10 and IL-4 would act as feed-back, trying to reduce these levels of pro-inflammatory cytokines, although with little success, possibly because the mechanisms that are producing these high levels of inflammatory molecules are very powerful and difficult to counteract. IL-10 is one of the most powerful anti-inflammatory cytokines and is involved in various regulatory actions of the immune and inflammatory systems (38). Some *in vitro* and *in vivo* studies have suggested that IL-10 could be used as a helpful therapeutic agent in the treatment of chronic and acute inflammatory processes, both systemic and localized (39). This cytokine has important suppressive properties in macrophages, T-cells and B cells (40). Both IL-10 and IL-4 have an important regulatory role in the cytokine network, acting as anti-inflammatory regulators in immune reactions in patients with CHF (37, 41). Curiously, we found a discrepancy between the levels detected in serum and the basal expression of mRNA, this could be due to an increased consumption of the cytokine in more inflammatory environments, or even to its blockage with the soluble form of its receptor (38).

The relationship between CMV and the host’s immune system is very intimate and produces multiple changes in the lymphocyte compartment. This virus takes advantage of the host’s inflammatory response to perpetuate itself and avoid being eliminated (42). In immunocompetent people, CMV is an asymptomatic, latent infection with periodic reactivations, whereas in immunosuppressed patients it usually causes acute pathology (43). The host’s inflammatory response is essential for the reactivation of CMV and is very important in stimulating the gene expression of the virus (44). In turn, some of the virus gene products positively regulate the production by the host of a wide variety of pro-inflammatory mediators (IL-1β, IL-6, TNF) (45). Moreover, highly differentiated T-lymphocytes could be being activated by agents implicated in chronic infections, and this activation would lead to increased cytokine production and possible tissue damage. CMV reactivations could be producing this continuous activation of highly differentiated T-lymphocytes, exacerbating cardiac pathology and the defective response of these activated lymphocytes. Immunosuppressed individuals, as is well known, can suffer dire consequences in the context of a CMV reactivation. As is already known in the elderly, CMV reactivations in CHF patients may not give any kind of symptoms and may go completely unnoticed, despite being quite frequent (46). The greater lymphocyte differentiation found in patients with CHF may partly explain the higher production and concentration of pro-inflammatory cytokines in these patients, since the more differentiated lymphocytes are producers of large amounts of inflammatory products (12, 47, 48).

On the other hand, CMV seropositivity has recently been associated with gut damage and microbial translocation, markers of intestinal damage have been associated with IgG levels against CMV in elderly patients and this has been associated with increased inflammation (49). The translocation of microbial products into circulation further contributes to systemic immune activation. Microbial translocation was first described by quantifying levels of the bacterial lipopolysaccharide (LPS) in blood circulation. It has been demonstrated that plasma levels of a fungal cell wall component are also elevated and related to inflammation (50). It would be of great interest to be able to study these parameters in our patients and see if there is any relationship between gut damage or microbial translocation and inflammation and worse functional status.

It has been known for years that the immune response in patients with CHF is shifted towards the Th1 pathway (9) and in our study we have shown that this response is even more exacerbated in CMV+ patients, possibly as a control to the possible reactivations of the virus, with an increased expression of TBX21 and IFN-γ (51). The increased expression of the pro-inflammatory molecules IL-8 and CCL2 can be explained in the context of chronic inflammatory disease, these molecules producing an attraction for monocytes, neutrophils and lymphocytes, involved in the inflammatory state of the pathology. This infection control could also be involved in the increased expression of genes associated with the activation of CD4+ T-lymphocytes, since both CMV surveillance and the continuous presence of pro-inflammatory cytokines in the environment may be continuously activating these lymphocytes. CMV reactivations leads to an increase in CD4+CD28null T-cells (52, 53). One of the main characteristics of this lymphocyte population is the production of cytotoxic molecules, such as perforin and granzyme B (47). In fact, we have found genes involved in cytotoxicity, such as perforin, granzyme B, and granulysin, overexpressed in CD4+ T-lymphocytes. Another characteristic of this CD4+CD28null population is its resistance to apoptosis (54, 55) this is corroborated by the increased expression of the BCL2 and BCL2L1 genes, antiapoptotic molecules, in CHF CMV+ patients. As a whole, we can affirm that CHF CMV+ patients compared with CMV- patients present an expression profile in CD4+ T-lymphocytes Th1 type, more activated and with a highly differentiated and highly reactive CD4+CD28null T population, that is possibly the result of the own fight against CMV infection and the environment, even more pro-inflammatory than in CMV- patients.

In summary, the high levels of pro-inflammatory cytokines found in CHF patients are due, as was already known, to the processes present in the context of CHF but are also, and in a very important way, related to dynamics of CMV-infection, since these high levels of cytokines are related to anti-CMV antibody titers and not only to CMV-infection. The inflammation found and the consequent immunosuppression are probably the main causes of the re-emergences of CMV, and the great lymphocyte differentiation demonstrated in CHF patients. Both characteristics are enhanced in patients with worse functional status, probably due to the negative effects of the chronic inflammation present in these patients.

## Data Availability Statement

The original contributions presented in the study are included in the article/**Supplementary Material**. Further inquiries can be directed to the corresponding authors.

## Ethics Statement

The studies involving human participants were reviewed and approved by Ethics committee of the Hospital Central de Asturias. The patients/participants provided their written informed consent to participate in this study.

## Author Contributions

The authors’ responsibilities were as follows–RA-A and MM-G: designed the study. AG-T, EB-G, RL-M, BR-B, and CQ: prepared protocols, collected and processed all the samples, performed or oversaw the experimental protocols, and analyzed data. AG-T and RA-A: wrote the manuscript. SA-A, BD-M and JLL: selected, recruited and followed up volunteers. MM-G and RA-A reviewed the manuscript. All authors contributed to the article and approved the submitted version.

## Funding

This research was supported by grant PI17/00714 from the Spanish I+D+i 2013–2016 State Program, which was cofounded by Instituto de Salud Carlos III and the European Regional Development Fund (ERDF).

## Conflict of Interest

The authors declare that the research was conducted in the absence of any commercial or financial relationships that could be construed as a potential conflict of interest.

## Publisher’s Note

All claims expressed in this article are solely those of the authors and do not necessarily represent those of their affiliated organizations, or those of the publisher, the editors and the reviewers. Any product that may be evaluated in this article, or claim that may be made by its manufacturer, is not guaranteed or endorsed by the publisher.
