# Supplementary material for: CMV Infection Is Directly Related to the Inflammatory Status in Chronic Heart Failure Patients
Source: Front Immunol. 2021 Aug 12;12:687582. doi: 10.3389/fimmu.2021.687582 (PMC8387659; doi:10.3389/fimmu.2021.687582)
Supplement: Supplementary file 1 [file Presentation_1.ppt]

## Slide 1
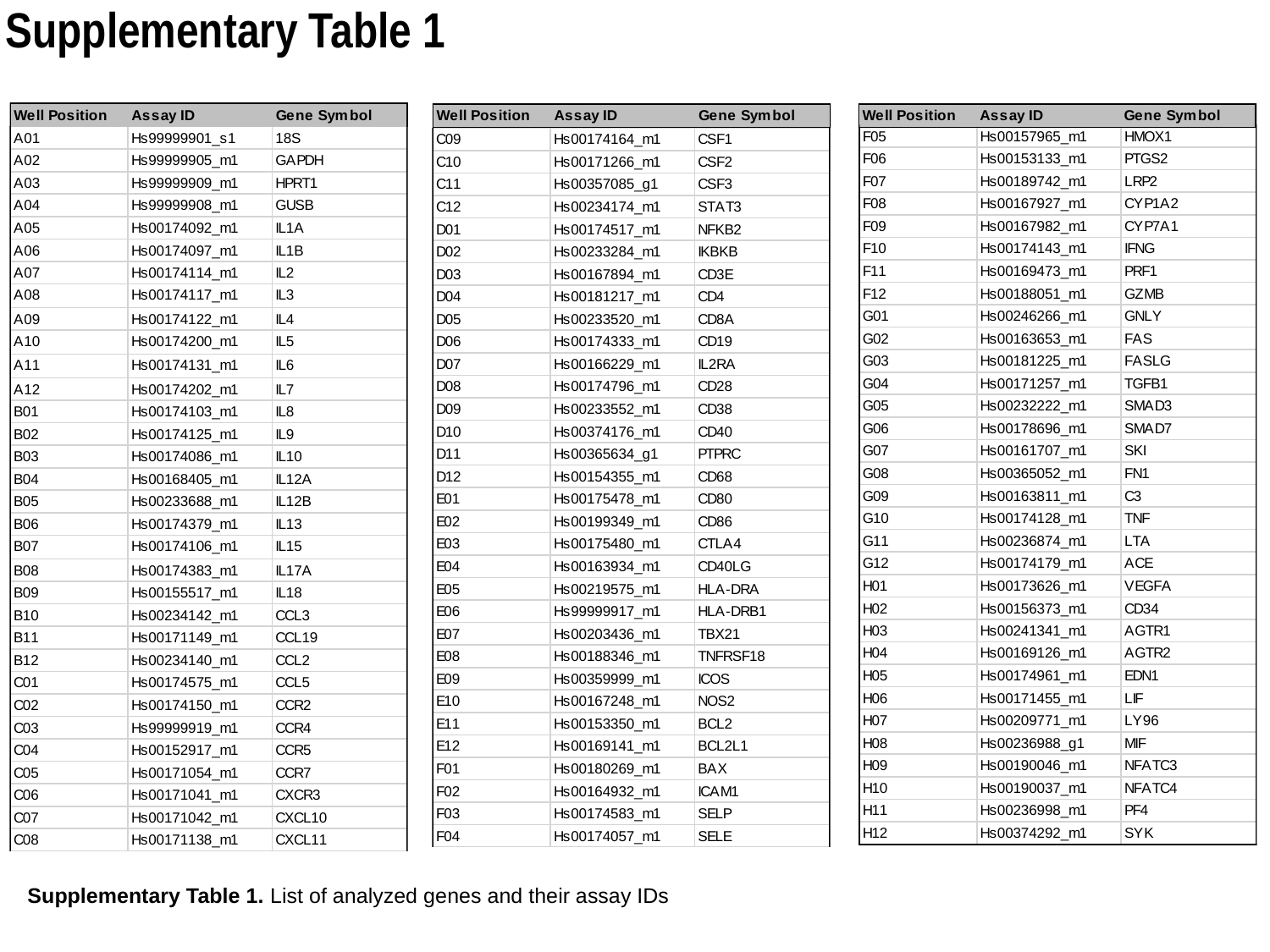

Supplementary Table 1
Supplementary Table 1. List of analyzed genes and their assay IDs

## Slide 2
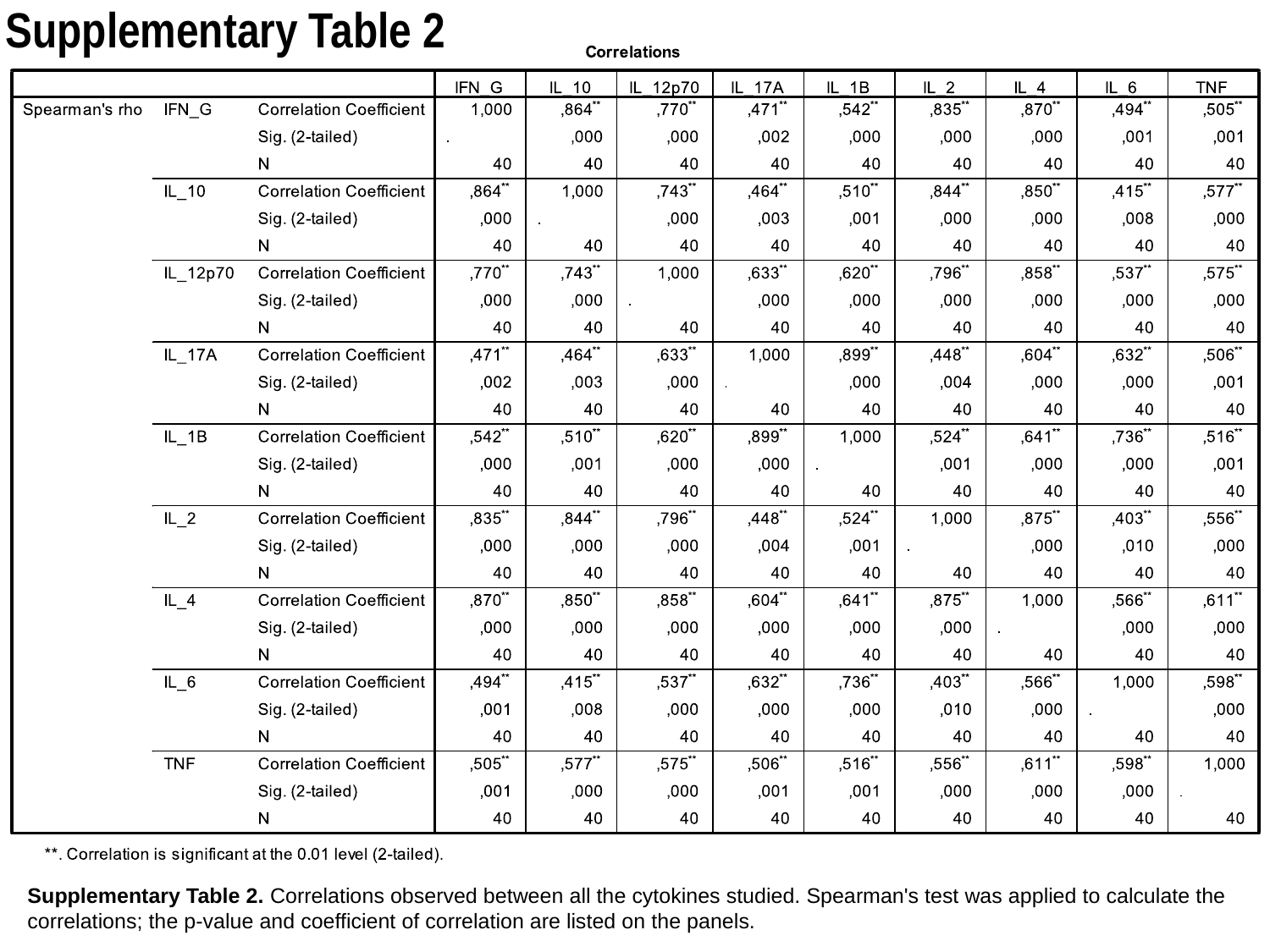

Supplementary Table 2
Supplementary Table 2. Correlations observed between all the cytokines studied. Spearman's test was applied to calculate the correlations; the p-value and coefficient of correlation are listed on the panels.

## Slide 3
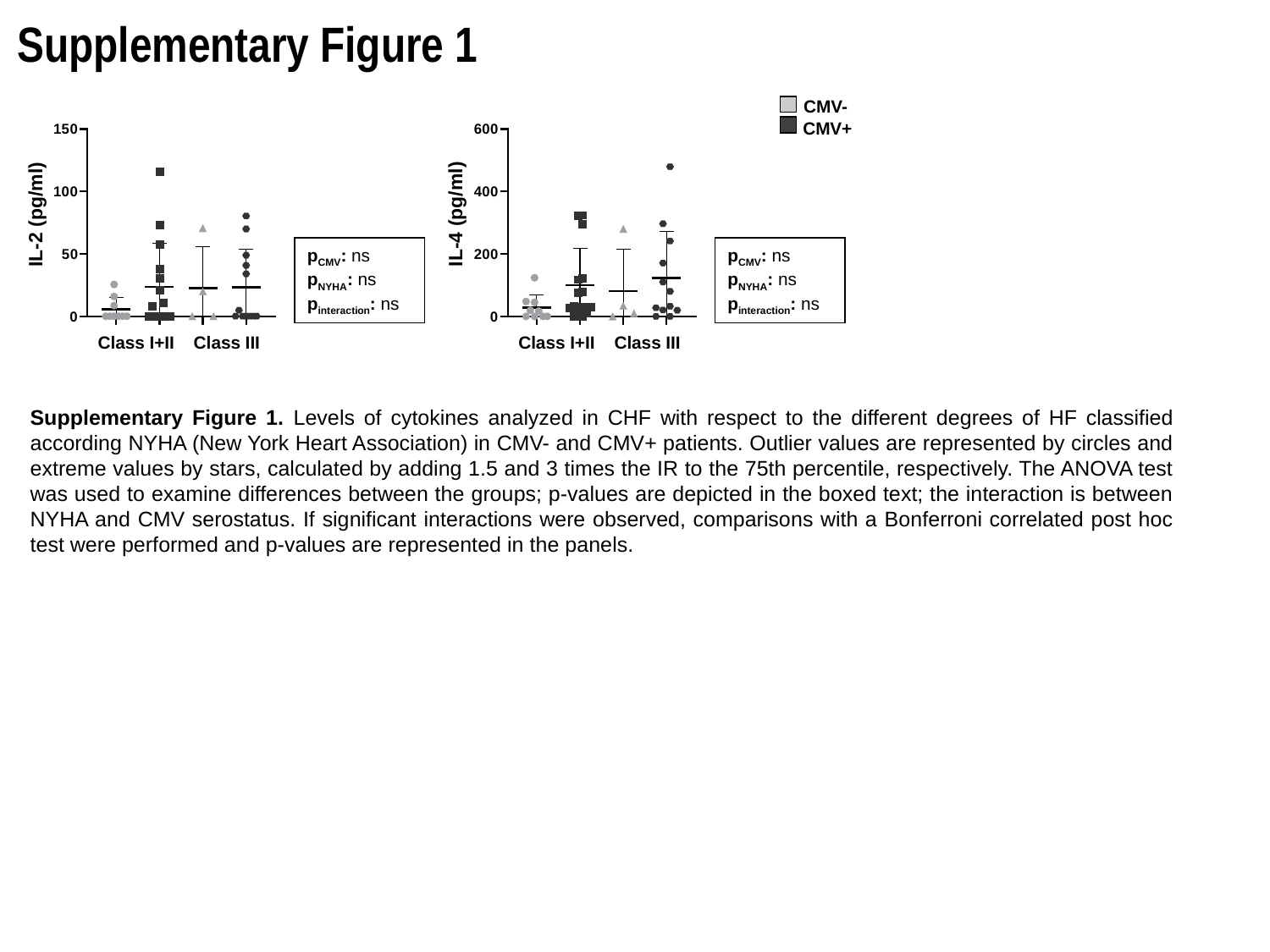

Supplementary Figure 1
 CMV-
 CMV+
IL-4 (pg/ml)
IL-2 (pg/ml)
pCMV: ns
pNYHA: ns
pinteraction: ns
pCMV: ns
pNYHA: ns
pinteraction: ns
Class I+II Class III
Class I+II Class III
Supplementary Figure 1. Levels of cytokines analyzed in CHF with respect to the different degrees of HF classified according NYHA (New York Heart Association) in CMV- and CMV+ patients. Outlier values are represented by circles and extreme values by stars, calculated by adding 1.5 and 3 times the IR to the 75th percentile, respectively. The ANOVA test was used to examine differences between the groups; p-values are depicted in the boxed text; the interaction is between NYHA and CMV serostatus. If significant interactions were observed, comparisons with a Bonferroni correlated post hoc test were performed and p-values are represented in the panels.

## Slide 4
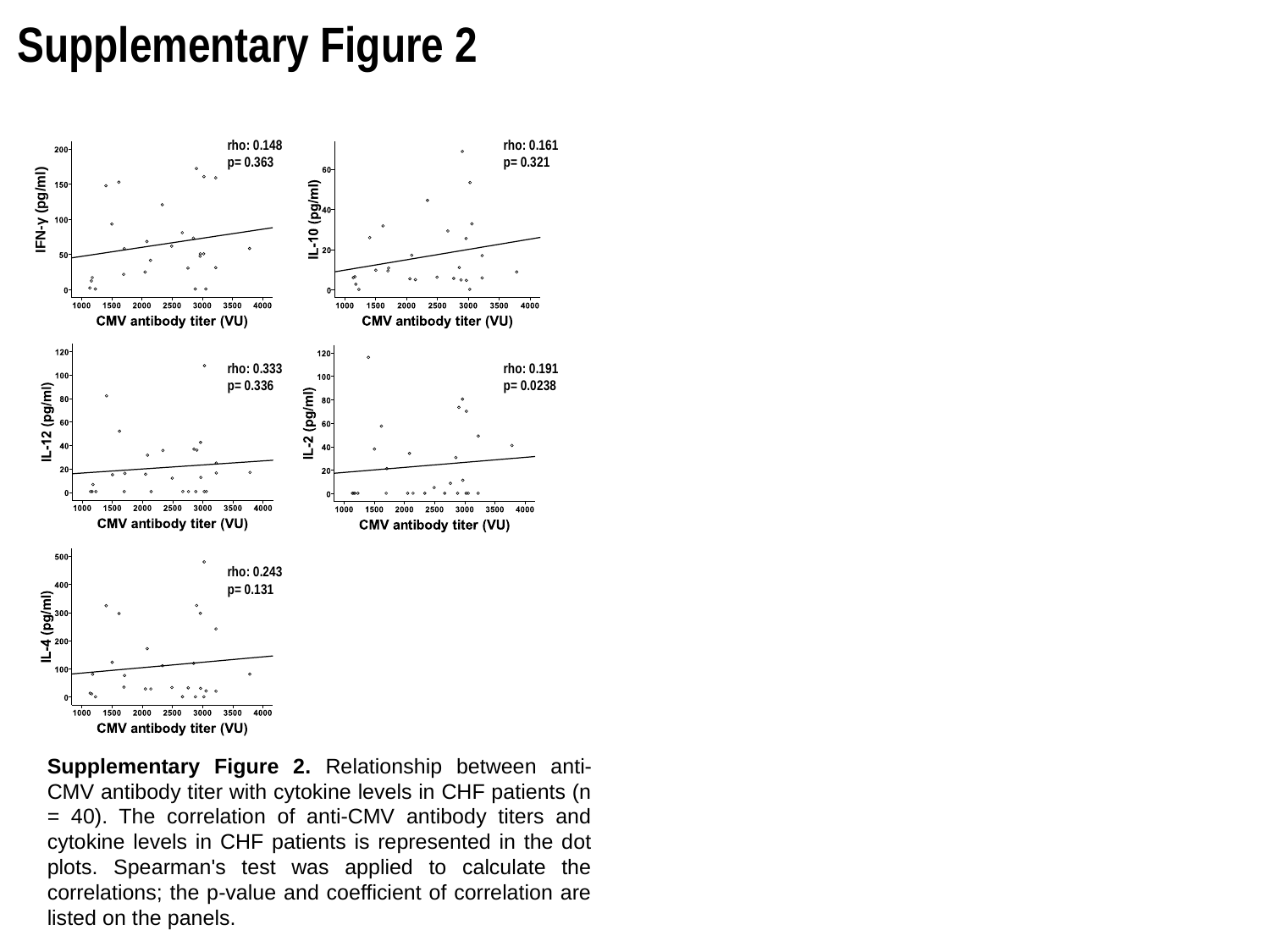

Supplementary Figure 2
IFN-γ (pg/ml)
rho: 0.148
p= 0.363
rho: 0.161
p= 0.321
rho: 0.333
p= 0.336
rho: 0.191
p= 0.0238
rho: 0.243
p= 0.131
Supplementary Figure 2. Relationship between anti-CMV antibody titer with cytokine levels in CHF patients (n = 40). The correlation of anti-CMV antibody titers and cytokine levels in CHF patients is represented in the dot plots. Spearman's test was applied to calculate the correlations; the p-value and coefficient of correlation are listed on the panels.

## Slide 5
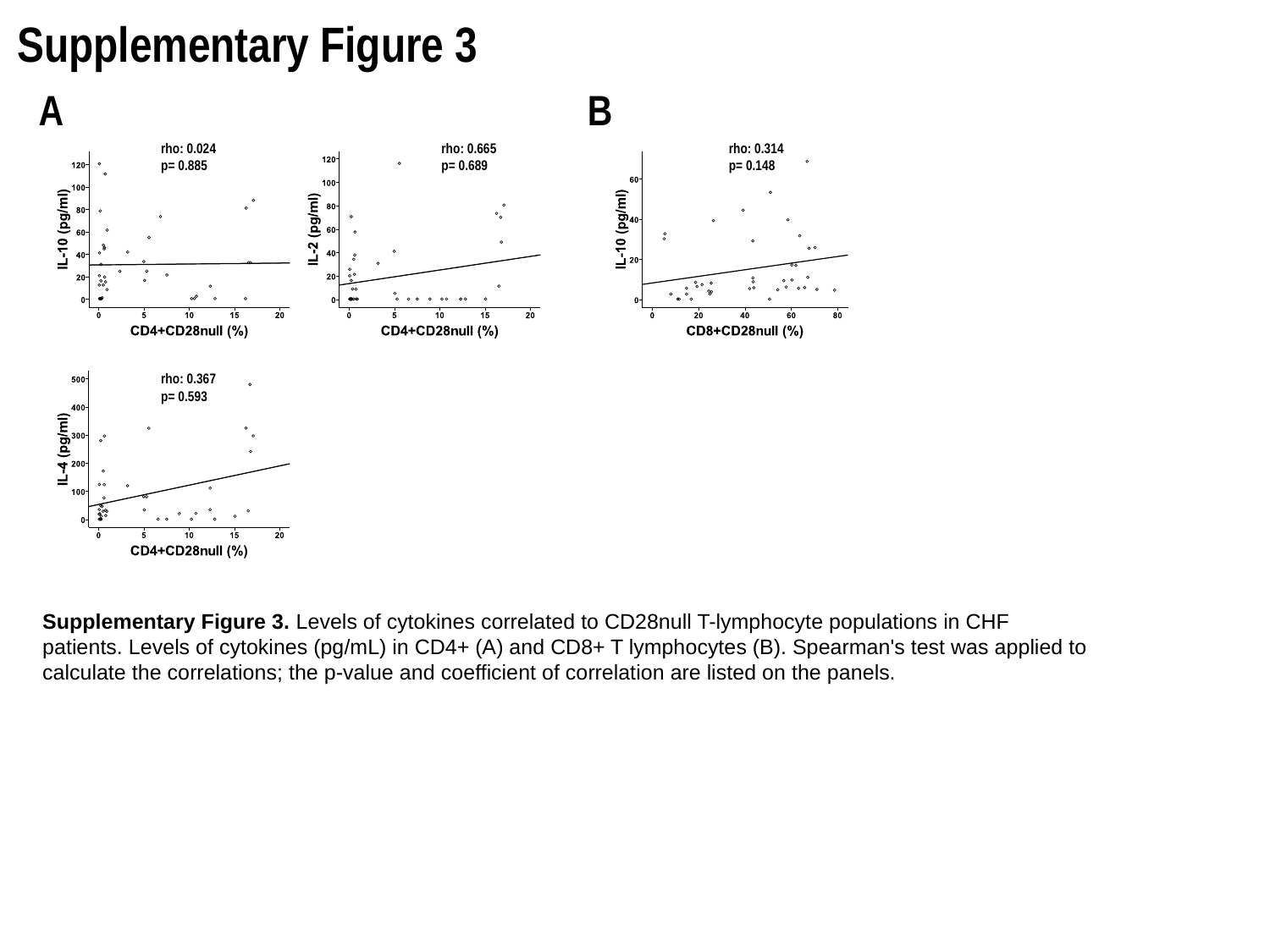

Supplementary Figure 3
A
B
rho: 0.024
p= 0.885
rho: 0.665
p= 0.689
rho: 0.314
p= 0.148
rho: 0.367
p= 0.593
Supplementary Figure 3. Levels of cytokines correlated to CD28null T-lymphocyte populations in CHF patients. Levels of cytokines (pg/mL) in CD4+ (A) and CD8+ T lymphocytes (B). Spearman's test was applied to calculate the correlations; the p-value and coefficient of correlation are listed on the panels.
